# Characteristics of a Surgical Snare Using Microwave Energy

**DOI:** 10.3390/diagnostics8040083

**Published:** 2018-12-15

**Authors:** Masashi Sugiyama, Kazuyuki Saito

**Affiliations:** 1 Graduate School of Science and Engineering, Chiba University, Chiba 263-8522, Japan; 2 Center for Frontier Medical Engineering, Chiba University, Chiba 263-8522, Japan; saito@faculty.chiba-u.jp

**Keywords:** microwave, EMR, snare, numerical calculation

## Abstract

Currently, minimally invasive treatments that insert various treatment devices into an endoscope are actively being performed. A high-frequency (HF) snare is commonly used as an energy device inserted into an endoscope. However, using a high-frequency snare, problems usually occur, such as the obstruction of the visual field caused by smoke. On the other hand, microwave heating produces less smoke and provides a better visual field. In this study, a snare using microwave energy inserted into an endoscope is proposed, and its characteristics are evaluated.

## 1. Introduction

In recent years, electromagnetic field techniques have been widely used in medical applications. Examples of these applications are microwave hyperthermia [1], microwave coagulation therapy used for liver cancer [2,3], cardiac catheter ablation for ventricular arrhythmia [4], and hyperthermia treatment for benign prostatic hyperplasia. These technologies are used to simulate the thermal effect of living tissue by the electromagnetic field.

**Figure 1:**
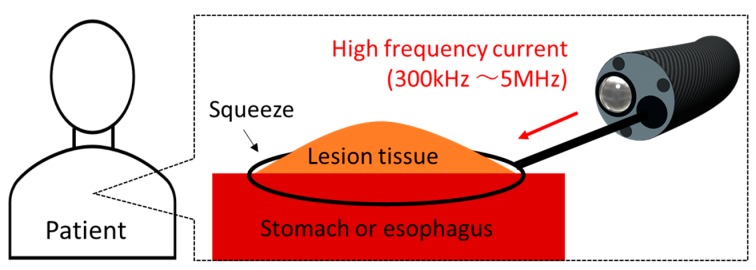
Schematic diagram of endoscopic mucosal resection (EMR).

One of the applications of the thermal effect is endoscopic mucosal resection (EMR) [5]. A schematic diagram of EMR is shown in Figure 1. EMR is mainly used for lesions of the stomach and the esophagus. The medical doctor inserts the endoscope into the mouth of the patient, and a snare is inserted into the forceps channel of the endoscope. The snare diameter can be changed to a certain extent. The doctor can put the snare on the location of the lesion and then squeeze and heat the lesion with the snare. An image of the surgery being performed can be viewed from a video monitor. The doctor can remove the lesion areas while stopping bleeding. EMR is performed at various medical institutions, and many cases have been reported [6,7]. However, as the current snare works at high-frequency (HF) currents (300 kHz to 5 MHz), the tissue will be carbonized, and smoke will be generated because of the excessively high temperature. The occurrence of perforations is also reported [8]. In addition, to the best of the author’s knowledge, there are no studies on snare development.

In this study, we designed an EMR snare using microwave energy. Microwave heating is derived from the vibration of water molecules. There are three advantages of using microwave energy. First, it has high tissue coagulation ability. Second, it does not generate smoke at the time of surgery because of mild heating. Third, tissue coagulation can be performed even under liquid conditions. With the use of HF currents, such currents are dispersed in the liquid, so the heating capability is lowered. By contrast, microwave energy does not have these limitations. For these reasons, microwave snares are considered to improve the quality of treatment.

In this study, the heating characteristics of a high frequency snare and a microwave snare are examined by numerical analysis and in vivo experiments.

## 2. Materials and Methods

### 2.1. Device Structure 

**Figure 2:**
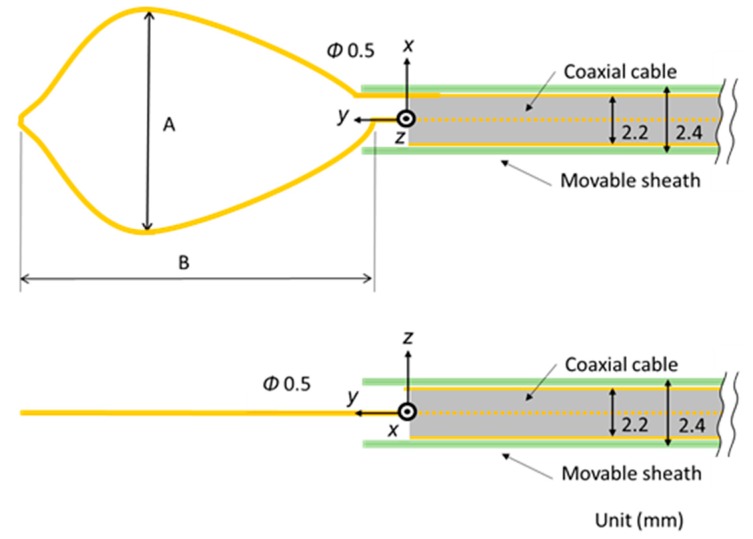
Schematic diagram of the proposed microwave snare.

**Figure 3:**
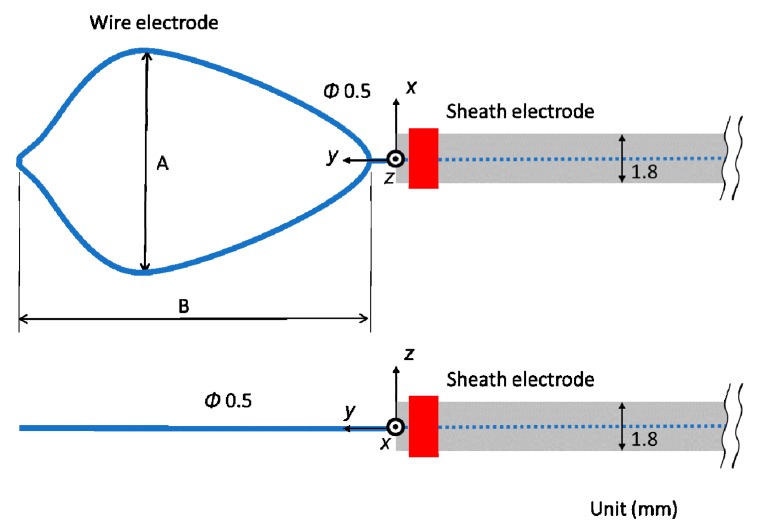
Schematic diagram of a commercially available snare.

Figure 2 shows the schematic diagram of the proposed microwave snare. It consists of a coaxial cable and a connecting wire. The inner and outer conductors are connected, and the connecting wire configures the main body of the snare. The coaxial cable is covered with a movable heath. This device operates like a loop antenna by exciting microwave energy from the end of the coaxial cable. The target part is grabbed and heated. Then, the snare is tightened while heating, and the target part can be removed. Figure 3 shows a schematic diagram of a commercially available snare. This snare consists of a wire electrode and a sheath electrode. The HF current runs between these two electrodes and causes Joule heating to be generated in the target tissue.

### 2.2. Electromagnetic Field Analysis

**Figure 4:**
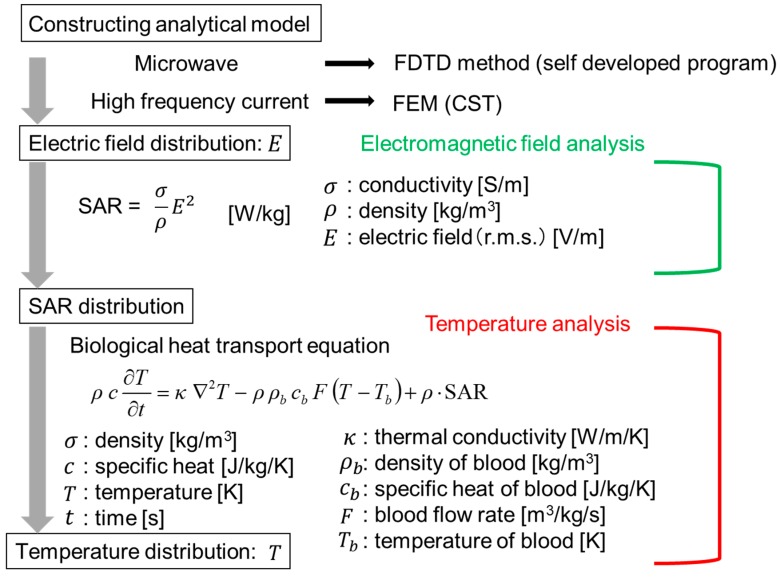
Numerical analysis procedure. FE—finite element.

The numerical analysis is shown in Figure 4. Analysis of the snare using microwave energy was performed with a self-developed program by utilizing the finite difference time domain (FDTD) method [9]. In addition, the HF current was analyzed with the finite element method (FEM) of CST (Computer Simulation Technology) EM Studio 2018. As FEM performs analysis in the frequency domain, this calculation takes time in the HF region. However, FEM is better than the FDTD method in the low-frequency region because it takes time for the signal to decay in the FDTD method. The electric field distribution near the device is first calculated.

Then, the specific absorption rate (SAR) in the biological tissue is determined. The SAR can be calculated by Equation (1):

(1) $$\mathrm{SAR}=\;\frac{\sigma }{\rho }E^{2}$$

In this equation, *σ* is the conductivity (S/m), *ρ* is the density (kg/m^3^), and *E* is the electric field (V/m) (r.m.s). Using this calculated SAR distribution as the heat source, the temperature distribution can be determined by solving the bioheat transfer equation [10]. The analytical parameters are shown in Table 1. The values in Table 1 are set considering realistic use at each energy. Therefore, the powers and the time of the “microwave” and the “HF” are not the same. In the HF snare, electric current flows directly to the living tissue, so its power is smaller than that of the microwave snare.

The analytical model is assumed to be used in EMR, and the situation is simulated, where the snare is used in stomach tissue on air. To simulate the grasping process of stomach tissue with the snare, a grasped stomach tissue is protruded. Three patterns of the length of the exposed snare are calculated to consider the squeezing process.

For each model, the electric field is obtained by inputting a voltage of 2.45 GHz for the microwave and 500 kHz for the HF voltage from the end of the cable to calculate the SAR distributions. Other electrical parameters are shown in Table 2 [11].

### 2.3. Temperature Analysis

The bioheat transfer equation used to obtain the temperature distribution of the living tissue is shown in Equation (2).

(2) $$\rho c\frac{\partial T}{\partial t}\;=\kappa \nabla^{2}T-\rho \rho_{b}c_{b}F\left(T-T_{b}\right)+\rho \cdot \mathrm{SAR}$$

*T* is the temperature and *t* is the time in the equation. *ρ* is the density (kg/m^3^) of the tissue and *ρ_b_* is the density of blood. *c* is the specific heat (J/kg/K) of the tissue and *c_b_* is the specific heat of blood. κ is the thermal conductivity (W/m/K), and *F* is the blood flow rate (m^3^/kg/s). The first term on the right side of this equation shows the diffusion of heat in the living body, the second term shows the dispersion of heat by blood flow, and the third term shows the heat generation source in the living body. The initial temperatures of the tissue, blood, and air are all 37 °C. Thermal constants are shown in Table 3 [11]. When the temperature reaches about 100 °C by microwave heating, moisture evaporates, so no further temperature increase occurs. Therefore, in the temperature analysis of microwave energy, the maximum temperature is limited to 100 °C. On the other hand, the temperature rises abruptly, and the tissue will be carbonized with the use of a HF current. An increase in temperature of 100 °C or more is considered. Therefore, the temperature was not limited.

## 3. Results

### 3.1. Calculated Results

**Figure 5:**
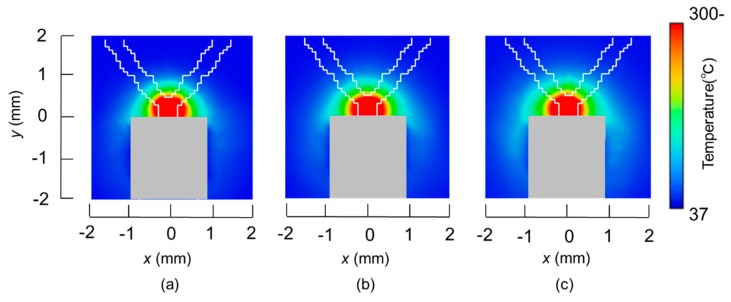
Temperature distributions of the high frequency snare at *z* = −0.3 mm.

**Figure 6:**
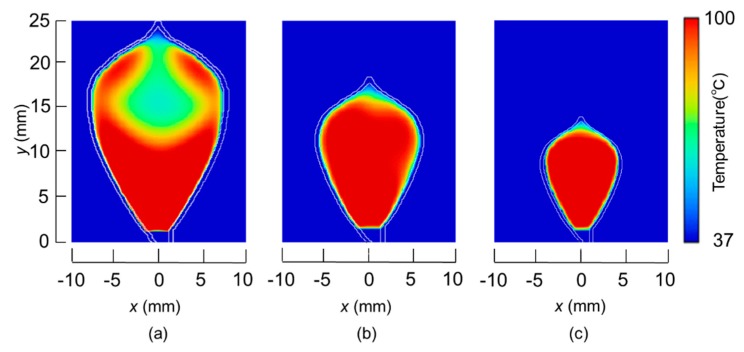
Temperature distributions of the microwave snare at *z* = −0.3 mm.

**Figure 7:**
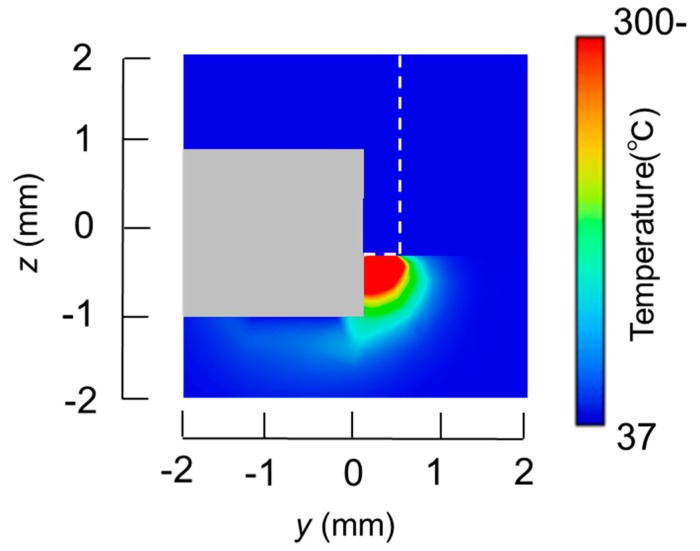
Temperature distributions of the high frequency snare at *x* = 0 mm.

**Figure 8:**
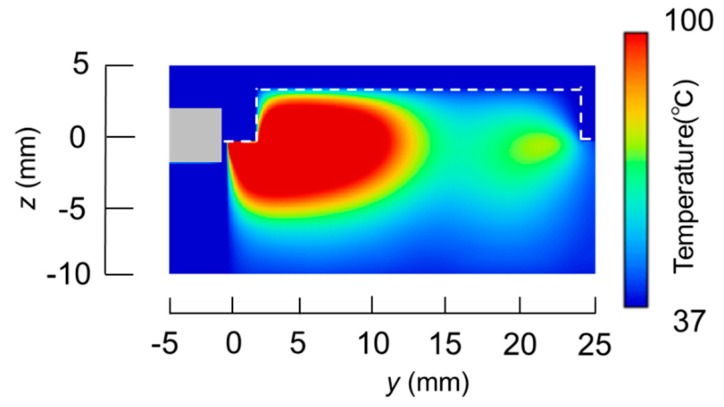
Temperature distribution of the microwave snare at *x* = 0 mm.

**Figure 9:**
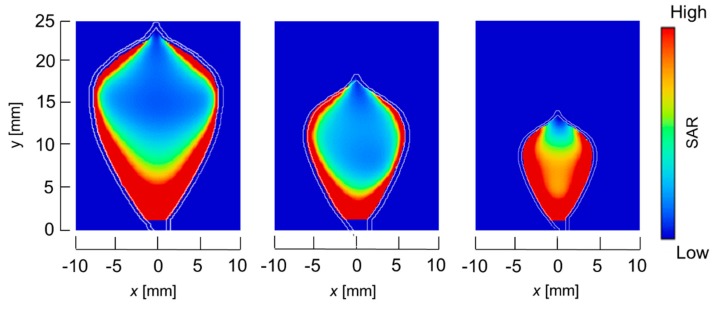
SAR distribution of the microwave snare at *z* = −0.3 mm.

The temperature distributions at the *xy* plane at *z* = −0.3 mm are shown in Figure 5 when the snare using a HF current is utilized. These are also shown in Figure 6 when the microwave snare is used. The observation surface in Figure 6 is set to be the same as that for a HF current. Regarding the exposed part’s length, (a), (b), and (c) represent 1/2 λ, 3/8λ, and 1/4λ, respectively. λ represents a wavelength of 2.45 GHz microwave energy in stomach tissue. Table 4 shows the relationship between the total length and the size of the snare. The lengths A and B are shown in Figure 2 and Figure 3, respectively. The white lines in Figure 5 and Figure 6 indicate snare outlines. In addition, cross sections of *x* = 0 when the snare’s exposed part length is 1/2λ are shown in Figure 7 and Figure 8. The white lines in Figure 7 and Figure 8 indicate the boundary between the stomach and air. In Figure 5, Figure 6, Figure 7 and Figure 8, the device body is grayed out because this part is excluded from the temperature evaluation. In Figure 5, high temperatures are observed at the *xy* plane (*z* = −0.3 mm) at *x* = *y* = 0 in all cases. This is the root of the snare. It can be inferred that perforation is caused by this localized heating. Compared with the temperature distribution in Figure 5a–c), it can be estimated that most of the current is concentrated at the root of the snare, so there is no difference in temperature distribution for different snare lengths. On the other hand, in the microwave snare, the entire gripping part is heated in Figure 6. This is advantageous for tissue removal by the snare. From Figure 7 and Figure 8, the maximum tissue coagulation depth over 60 °C, which is the tissue coagulation temperature, is 1.5 mm and 5.8 mm with the HF current and the microwave energy, respectively. Because microwave energy heats to a greater depth than the HF current does, caution may be required in clinical application. The SAR distribution of the microwave snare is shown in Figure 9. In this figure, a high SAR is observed at the vicinity and root of the snare. Therefore, the microwave snare can be heated regardless of the shape of the snare.

### 3.2. Experimental Validation

**Figure 10:**
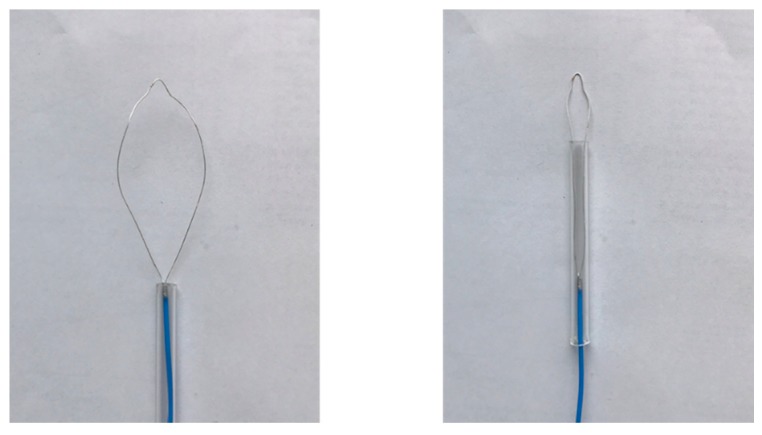
Prototype device.

**Figure 11:**
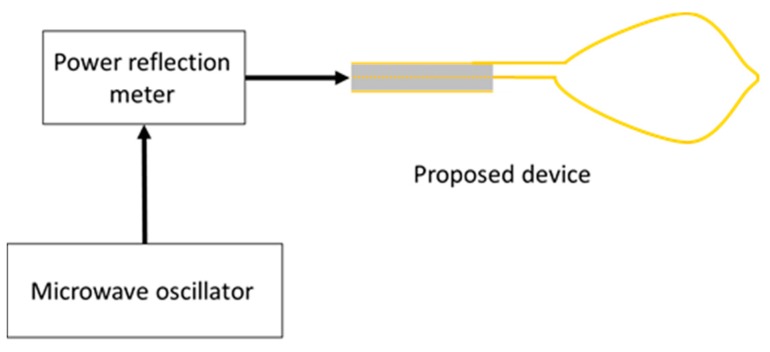
Experimental system.

Figure 10 shows an image of the prototype device. The dimensions of the device are the same as those of the numerical model. The snare is made of annealing copper. The device is connected to the experimental system, shown in Figure 11. The 2.45 GHz microwave energy is inputted from the microwave generator through the power reflection meter to the end of the prototype device. Porcine liver was used because it can be easily obtained in this experiment, and discoloration can be easily observed. The dielectric and thermal properties of porcine liver are similar to those of human liver, and there are no substantial differences in electrical properties between the stomach and the liver. The center of porcine liver is protruded to simulate the surgical condition of EMR. The protruding part is squeezed with a snare, coagulated, and removed by reducing the exposed diameter of the snare while heating. The input power is 58 W.

**Figure 12:**
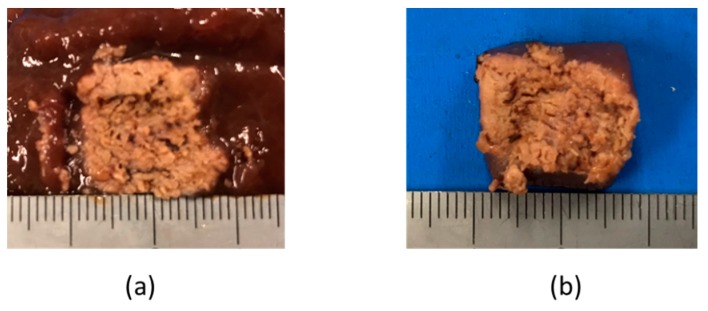
Liver tissue surface after the experiment.

**Figure 13:**
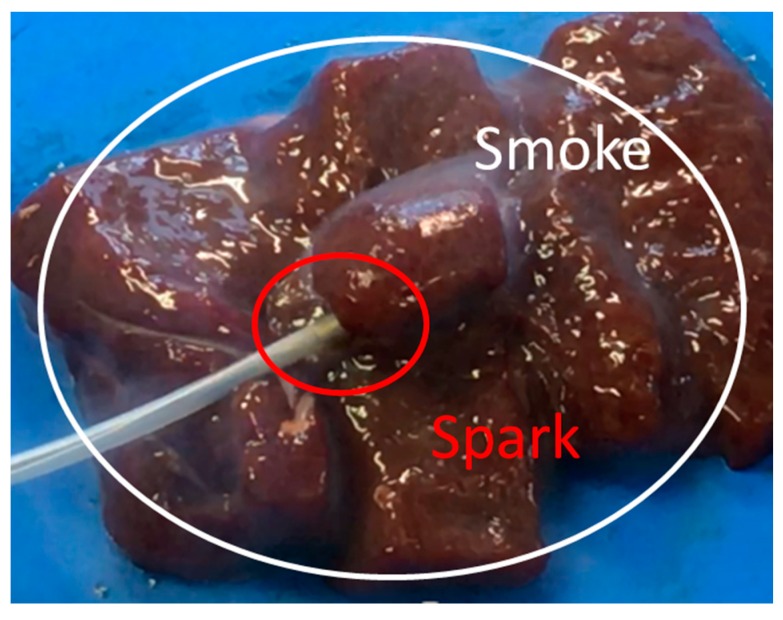
Heating with the high frequency current snare.

**Figure 14:**
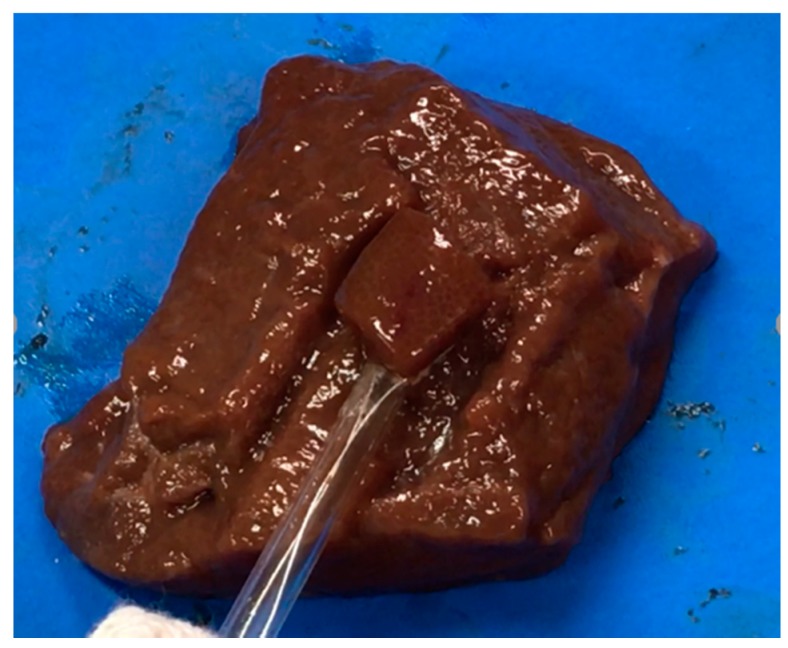
Heating with the microwave snare.

The liver tissue surface after the experiment is shown in Figure 12a, and the removed tissue is shown in Figure 12b. It can be confirmed that discoloration occurs in the entire gripping part, even in the center part, which is the furthest part from the snare. In addition, there is no blackened part in the discolored portion of the tissue. It can be concluded that the entire gripping part can be heat coagulated by the device, and the tissue can be heated without charring by the device. Figure 13 and Figure 14 show the overall view during the heating process. In Figure 13, during the HF current heating, smoke and sparks are observed. On the other hand, during the microwave heating, these are not observed (Figure 14).

## 4. Discussion

In this study, we proposed a snare using microwave energy for EMR. The effectiveness of this snare was evaluated by numerical analysis and a heating experiment. In the numerical analysis, analytical models were made for the HF current and the microwave, and the temperature distributions of the target part were obtained for comparison and evaluation. The temperature distribution was calculated by using the bioheat transfer equation with SAR as the heat source. The results confirm that sufficient heating for tissue coagulation was possible with the proposed device. From the heating experiment of the prototype device, it was also confirmed that the entire gripping part could be coagulated without carburization. We plan to develop devices that can be used for animal experimentation.

## Figures and Tables

**Table 1:** Analytical parameters.

Parameters	Microwave Snare	High Frequency Snare
Calculation method	FDTD	FEM
Power (W)	60	30
Heating time (s)	10	3

**Table 2:** Electrical constants.

Electrical Constants	Frequency	Stomach
Relative permittivity	500 kHz	2060
	2.45 GHz	43.0
Conductivity (S/m)	500 kHz	0.55
	2.45 GHz	1.69

**Table 3:** Thermal constants.

Thermal Constants	Objects	Values
Specific heat (J/kg/K)	Stomach	3690
	Fluororesin	1000
	Blood	3960
Thermal conductivity (W/m/K)	Stomach	0.53
	Fluororesin	0.23
Density (kg/m^3^)	Stomach	1088
	Fluororesin	2200
	Blood	1050
Blood flow rate (m^3^/kg/s)	Stomach	1.43 × 10^−5^

**Table 4:** Relationship between the total length and size of the snare.

Sizes	A (mm)	B (mm)
1/2λ	15	23
3/8λ	11	15
1/4λ	8	13
